# Supplementary material for: Hybrid Models and Biological Model Reduction with PyDSTool
Source: PLoS Comput Biol. 2012 Aug 9;8(8):e1002628. doi: 10.1371/journal.pcbi.1002628 (PMC3415397; doi:10.1371/journal.pcbi.1002628)
Supplement: Text S4 — Complete source code for the PyDSTool package (version 0.88.120504). Includes API documentation and help files linking to web pages. This file is identical to the current public release on Sourceforge.net. (ZIP) [file pcbi.1002628.s004.zip › PyDSTool/html/PyDSTool.Toolbox.optimizers.tests.test_quadratic.test_Quadratic-class.html]

xml version="1.0" encoding="ascii"?


PyDSTool.Toolbox.optimizers.tests.test\_quadratic.test\_Quadratic


| Home | Trees | Indices | Help | PyDSTool |
| --- | --- | --- | --- | --- |

Package PyDSTool ::
Package Toolbox ::
Package optimizers ::
Package tests ::
Module test\_quadratic ::
Class test\_Quadratic

[hide private]

[frames] | no frames]

# Class test\_Quadratic

source code

```
??-102 --+
         |
        test_Quadratic
```

---

Global test class with a quadratic function


|  |  |
| --- | --- |
| Instance Methods | [hide private] |

|  |  |
| --- | --- |
| check\_simple\_gradient(self) | source code |

|  |  |
| --- | --- |
| check\_simple\_newton(self) | source code |

|  |  |
| --- | --- |
| check\_damped\_gradient\_relative(self) | source code |

|  |  |
| --- | --- |
| check\_swp\_frgradient\_relative(self) | source code |

|  |  |
| --- | --- |
| check\_swp\_cwgradient\_relative(self) | source code |

|  |  |
| --- | --- |
| check\_swp\_prpgradient\_relative(self) | source code |

|  |  |
| --- | --- |
| check\_swp\_dpgradient\_relative(self) | source code |

|  |  |
| --- | --- |
| check\_swp\_dypgradient\_relative(self) | source code |

|  |  |
| --- | --- |
| check\_simple\_marquardt(self) | source code |

| Home | Trees | Indices | Help | PyDSTool |
| --- | --- | --- | --- | --- |

|  |  |
| --- | --- |
| Generated by Epydoc 3.0.1 on Fri May 4 15:24:09 2012 | http://epydoc.sourceforge.net |
